# Supplementary material for: Computational pipeline to probe NaV1.7 gain-of-function variants in neuropathic painful syndromes
Source: Sci Rep. 2020 Oct 21;10:17930. doi: 10.1038/s41598-020-74591-y (PMC7578092; doi:10.1038/s41598-020-74591-y)
Supplement: Supplementary file 1 — Supplementary Information. [file 41598_2020_74591_MOESM1_ESM.pdf]

Supplementary Material to: Computational Pipeline to probe NaV 1.7 gain-of-function variants in neuropathic painful syndromes

Alberto A. Toffano[1], Giacomo Chiarot[2], Stefano Zamuner[3], Margherita Marchi[4], Erika Salvi[4], Stephen G. Waxman[5], Catharina G. Faber[6,7], Giuseppe Lauria[4,8], Achille Giacometti[1,9], and Marta Simeoni[2,9,*]

[1]Universitá Ca' Foscari Venezia, Dipartimento di Scienze Molecolari e Nanosistemi, Venezia-Mestre, Italy
[2]Universitá Ca' Foscari Venezia, Dipartimento di Scienze Ambientali, Informatica e Statistica, Venezia-Mestre, Italy
[3]Ècole Polytechnique Fèdèrale de Lausanne (EPFL), Laboratory of Statistical Biophysics, Institute of Physics, School of Basic Sciences, Lausanne, Switzerland
[4]Fondazione IRCCS Istituto Neurologico "Carlo Besta", Neuroalgology Unit, Milan, Italy
[5]Center for Neuroscience and Regeneration Research, VA Connecticut Healthcare System, West Haven, USA
[6]Maastricht University, MHeNs school for Mental Health and Neuroscience, Maastricht, The Netherlands
[7]Maastricht University Medical Center, Department of Neurology, Maastricht, The Netherlands
[8]University of Milan, Department of Biomedical and Clinical Sciences "Luigi Sacco", Milan, Italy
[9]European Centre for Living Technology (ECLT), Venice, Italy
[*]simeoni@unive.it

1 Variants List

Table S1 shows the list of mutations of the **PAT** group and their characteristics. Table S2 shows the same information for the **NEUTRAL** group.

**ID**	**Mutation**	**Group**	**gpos**	**cpos**	**rsID**	**Freq**
0	I136V	IEM	2:167163081	c.406A>G	rs80356468	
1	S211P	IEM	2:167160805	c.631T>C		
2	F216S	IEM	2:167160789	c.647T>C	rs80356469	
3	I234T	IEM	2:167159800	c.701T>C		
4	S241T	IEM	2:167159780	c.721T>A	rs80356470	0,00000
5	N395K	IEM	2:167145076	c.1185C>G	rs80356471	0,00002
6	V400M	IEM	2:167145063	c.1198G>A	rs1553491169	
7	L823R	IEM	2:167134666	c.2468T>G	rs80356473	0,000004
8	I848T	IEM	2:167133791	c.2543T>C	rs80356474	
9	L858H	IEM	2:167133761	c.2573T>A	rs80356475	0,00000
10	L858F	IEM	2:167133762	c.2572C>T	rs80356476	
11	A863P	IEM	2:167133747	c.2587G>C	rs80356477	0,000012
12	V872G	IEM	2:167133719	c.2615T>G		
13	P1308L	IEM	2:167085451	c.3923C>T		
14	V1316A	IEM	2:167085427	c.3947T>C		
15	F1449V	IEM	2:167083097	c.4345T>G	rs80356478	
16	W1538R	IEM	2:167060594	c.4612T>C	rs202084411	0,002051
17	A1746G	IEM	2:167055879	c.5237C>G		
18	V1298D	PEPD	2:167085481	c.3893T>A	rs121908911	0,00001
19	V1298F	PEPD	2:167085482	c.3892G>T	rs121908912	
20	V1299F	PEPD	2:167085479	c.3895G>T	rs121908913	
21	G1607R	PEPD	2:167056297	c.4819G>C		
22	M1627K	PEPD	2:167056236	c.4880T>A		
23	A1632E	PEPD	2:167056221	c.4895C>A	rs879253994	0,000004
24	R185H	SFN	2:167162344	c.554G>A	rs73969684	0,005294
25	I228M	SFN	2:167160752	c.684C>G	rs71428908	0,000574
26	I739V	SFN	2:167136962	c.2215A>G	rs182650126	0,002449
27	G856D	SFN	2:167133767	c.2567G>A	rs879254102	
28	M932L	SFN	2:167133540	c.2794A>C	rs12478318	0,036818
29	M1532I	SFN	2:167060610	c.4596G>A	rs200328637	0,000032
30	T1596I	PDN	2:167056329	c.4787C>T	rs200470541	0,000052

Table S1: List of deleterious mutations of the **PAT** group with related information and frequencies among individuals: **gpos** is the genomic position, **cpos** is the coding position, **rsID** is the genetic variation code as reported in dbSNP, **Freq** is the allele frequency in annotation databases.

ID	Mutation	Group	gpos	cpos	rsID	Freq
31	S126A	mammals				
32	L127A	mammals				
33	M145L	human	2:167163054	c.433A>T	rs949893512	0,000008
34	N146S	mammals				
35	V194I	mammals				
36	L201V	human	2:167160835	c.601T>G	rs80356465	0,000008
37	N206D	human	2:167160820	c.616A>G	rs80356466	
38	T370M	human	2:167145152	c.1109C>T	rs200391162	0,000409
39	E759D	mammals				
40	A766T	mammals				
41	A766V	human	2:167136880	c.2297C>T	rs749482425	0,00001
42	I767V	human	2:16713687	c.2299A>G	rs544352696	0,000016
43	T773S	human	2:167134816	c.2318C>G	rs200624920	0,000078
44	V795I	mammals				
45	A815S	mammals				
46	D890E	mammals				
47	D890V	mammals				
48	T920N	nABN	2:167133575	c.2759C>T	rs1299624986	0,000004
49	K1176R	mammals				
50	R1207K	human	2:167094752	c.3620G>A	rs267598973	0,00002
51	T1210N	mammals				
52	I1235V	mammals				
53	N1245S	nABN	2:167094638	c.3734A>G	rs141268327	0,004493
54	L1267V	nABN	2:167089942	c.3799C>G	rs180922748	0,001217
55	T1398M	human	2:167084214	c.4193C>T	rs200763228	0,000042
56	I1399D	mammals				
57	D1411N	mammals				
58	K1412E	mammals				
59	K1412I	mammals				
60	K1415I	mammals				
61	S1419N	human	2:167083186	c.4256G>A	rs201479177	0,000019
62	V1428I	nABN	2:167083160	c.4282G>A	rs149346064	0,001613
63	A1505V	human	2:167060692	c.4514C>T	rs779201398	0,000008
64	S1509A	mammals				
65	S1509T	mammals				
66	Q1530D	mammals				
67	Q1530K	human	2:167060618	c.4588C>T	rs1064796825	
68	Q1530P	mammals				
69	H1531Y	human	2:167060615	c.4591C>T	rs1337886698	0,000004
70	M1532V	human	2:167060612	c.4594A>G	rs201075910	
71	E1534D	mammals				
72	Y1537N	mammals				
73	T1548S	mammals				
74	H1560C	mammals				
75	H1560Y	mammals				
76	V1565I	mammals				
77	I1577L	mammals				
78	D1586E	human	2:167056358	c.4758T>G	rs1264810697	
79	T1590K	human	2:167056347	c.4769C>T	rs371341018	0,000016
80	T1590R	mammals				
81	V1613I	human	2:167056119	c.4836_4837delinsGA	rs1553473210	
82	V1662A	mammals				
83	G1674A	mammals				
84	K1700A	mammals				

Table S2: List of **NEUTRAL** variants with related information and frequencies among individuals: **gpos** is the genomic position, **cpos** is the coding position, **rsID** is the genetic variation code as reported in dbSNP, **Freq** is the allele frequency in annotation databases. Mammals: pseudo mutations identified among SCN9A homologous genes from mammalian species sharing > 90% nucleotide sequence identity. Human: genetic variants from dbSNPs with uncertain significance or benign that do not alter the biophysical properties of the channel.

2 Templates alignments

In this section we present the alignments of the WT sequence (NCBI code *NP_002968.1*) with the MOESM3, 6A90 and 6J8J templates sequences.

All the alignments have been performed by using Clustal Omega.

2.1 Alignment WT – MOESM3

In this section we present the alignment of the WT sequence with the MOESM3 template sequence. The sequence identity is 50.8%.

```
CLUSTAL O(1.2.4) multiple sequence alignment

WT              MAMLPPPGPQSFVHFTKQSLALIEQRIAERKSKEPKEEKKDDDEEAPKPSSDLEAGKQLP 60
MOESM3          ------------------------------------------------------------


WT              FIYGDIPPGMVSEPLEDLDPYYADKKTFIVLNKGKTIFRFNATPALYMLSPFSPLRRISI 120
MOESM3          ------------------------------------------------------------


WT              KILVHSLFSMLIMCTILTNCIFMTMNNPPDWTKNVEYTFTGIYTFESLVKILARGFCVGE 180
MOESM3          KILVHSLFSMLIMCTILTNCIFMTMNNPPDWTKNVEYTFTGIYTFESLVKILARGFCVGE 60
                ************************************************************

WT              FTFLRDPWNWLDFVVIVFAYLTEFVNLGNVSALRTFRVLRALKTISVIPGLKTIVGALIQ 240
MOESM3          FTFLRDPWNWLDFVVIVFAYLTEFVNLGNVSALRTFRVLRALKTISVIPGLKTIVGALIQ 120
                ************************************************************

WT              SVKKLSDVMILTVFCLSVFALIGLQLFMGNLKHKCFRNSLENNETLESIMNTLESEEDFR 300
MOESM3          SVKKLSDVMILTVFCLSVFALIGLQLFM-------------------------------- 148
                ****************************

WT              KYFYYLEGSKDALLCGFSTDSGQCPEGYTCVKIGRNPDYGYTSFDTFSWAFLALFRLMTQ 360
MOESM3          ----------------------------------------YTSFDTFSWAFLALFRLMTQ 168
                                                        ********************

WT              DYWENLYQQTLRAAGKTYMIFFVVVIFLGSFYLINLILAVVAMAYEEQNQANIEEAKQKE 420
MOESM3          DYWENLYQQTLRAAGKTYMIFFVVVIFLGSFYLINLILAVVAMAYEEQNQANIE------ 222
                ******************************************************

WT              LEFQQMLDRLKKEQEEAEAIAAAAAEYTSIRRSRIMGLSESSSETSKLSSKSAKERRNRR 480
MOESM3          ------------------------------------------------------------


WT              KKKNQKKLSSGEEKGDAEKLSKSESEDSIRRKSFHLGVEGHRRAHEKRLSTPNQSPLSIR 540
MOESM3          ------------------------------------------------------------


WT              GSLFSARRSSRTSLFSFKGRGRDIGSETEFADDEHSIFGDNESRRGSLFVPHRPQERRSS 600
MOESM3          ------------------------------------------------------------


WT              NISQASRSPPMLPVNGKMHSAVDCNGVVSLVDGRSALMLPNGQLLPEGTTNQIHKKRRCS 660
MOESM3          ------------------------------------------------------------


WT              SYLLSEDMLNDPNLRQRAMSRASILTNTVEELEESRQKCPPWWYRFAHKFLIWNCSPYWI 720
MOESM3          ------------------------------------------------------------


WT              KFKKCIYFIVMDPFVDLAITICIVLNTLFMAMEHHPMTEEFKNVLAIGNLVFTGIFAAEM 780
MOESM3          -----EYFIVMDPFVDLAITICIVLNTLFMAMEHHPMTEEFKNVLAIGNLVFTGIFAAEM 277
                      ******************************************************
```

```
WT          VLKLIAMDPYEYFQVGWNIFDSLIVTLSLVELFLADVEGLSVLRSFRLLRVFKLAKSWPT 840
MOESM3      VLKLIAMDPYEYFQVGWNIFDSLIVTLSLVELFLADVEGLSVLRSFRLLRVFKLAKSWPT 337
            ************************************************************

WT          LNMLIKIIGNSVGALGNLTLVLAIIVFIFAVVGMQLFGKSYKECVCKINDDCTLPRWHMN 900
MOESM3      LNMLIKIIGNSVGALGNLTLVLAIIVFIFAVVGMQLFGKSYKECVCKINDDCTLPRWHMN 397
            ************************************************************

WT          DFFHSFLIVFRVLCGEWIETMWDCMEVAGQAMCLIVYMMVMVIGNLVVLNLFLALLLSSF 960
MOESM3      DFFHSFLIVFRVLCGEWIETMWDCMEVAGQAMCLIVYMMVMVIGNLVVLNLFLALLLSSF 457
            ************************************************************

WT          SSDNLTAIEEDPDANNLQIAVTRIKKGINYVKQTLREFILKAFSKKPKISREIRQAEDLN 1020
MOESM3      SSDNLT------------------------------------------------------ 463
            ******

WT          TKKENYISNHTLAEMSKGHNFLKEKDKISGFGSSVDKHLMEDSDGQSFIHNPSLTVTVPI 1080
MOESM3      ------------------------------------------------------------


WT          APGESDLENMNAEELSSDSDSEYSKVRLNRSSSSECSTVDNPLPGEGEEAEAEPMNSDEP 1140
MOESM3      ------------------------------------------------------------


WT          EACFTDGCVRRFSCCQVNIESGKGKIWWNIRKTCYKIVEHSWFESFIVLMILLSSGALAF 1200
MOESM3      -----------------------------------KIVEHSWFESFIVLMILLSSGALAF 488
                                               *************************

WT          EDIYIERKKTIKIILEYADKIFTYIFILEMLLKWIAYGYKTYFTNAWCWLDFLIVDVSLV 1260
MOESM3      EDIYIERKKTIKIILEYADKIFTYIFILEMLLKWIAYGYKTYFTNAWCWLDFLIVDVSLV 548
            ************************************************************

WT          TLVANTLGYSDLGPIKSLRTLRALRPLRALSRFEGMRVVVNALIGAIPSIMNVLLVCLIF 1320
MOESM3      TLVANTLGYSDLGPIKSLRTLRALRPLRALSRFEGMRVVVNALIGAIPSIMNVLLVCLIF 608
            ************************************************************

WT          WLIFSIMGVNLFAGKFYECINTTDGSRFPASQVPNRSECFALMNVSQNVRWKNLKVNFDN 1380
MOESM3      WLIFSIMGVNLFAGKFYECINTTDGSRFPASQVPNRSECFALMNVSQNVRWKNLKVNFDN 668
            ************************************************************

WT          VGLGYLSLLQVATFKGWTIIMYAAVDSVNVDKQPKYEYSLYMYIYFVVFIIFGSFFTLNL 1440
MOESM3      VGLGYLSLLQVATFKGWTIIMYAAVDSVNVDKQPKYEYSLYMYIYFVVFIIFGSFFTLNL 728
            ************************************************************

WT          FIGVIIDNFNQQKKKLGGQDIFMTEEQKKYYNAMKKLGSKKPQKPIPRPGNKIQGCIFDL 1500
MOESM3      FIGVIIDNFNQQKKKLG-----------------------------------------DL 747
            *****************                                         **

WT          VTNQAFDISIMVLICLNMVTMMVEKEGQSQHMTEVLYWINVVFIILFTGECVLKLISLRH 1560
MOESM3      VTNQAFDISIMVLICLNMVTMMVEKEGQSQHMTEVLYWINVVFIILFTGECVLKLISLRH 807
            ************************************************************

WT          YYFTVGWNIFDFVVVIISIVGMFLADLIETYFVSPTLFRVIRLARIGRILRLVKGAKGIR 1620
MOESM3      YYFTVGWNIFDFVVVIISIVGMFLADLIETYFVSPTLFRVIRLARIGRILRLVKGAKGIR 867
            ************************************************************

WT          TLLFALMMSLPALFNIGLLLFLVMFIYAIFGMSNFAYVKKEDGINDMFNFETFGNSMICL 1680
MOESM3      TLLFALMMSLPALFNIGLLLFLVMFIYAIFGMSNFAYVKKEDGINDMFNFETFGNSMICL 927
            ************************************************************

WT          FQITTSAGWDGLLAPILNSKPPDCDPKKVHPGSSVEGDCGNPSVGIFYFVSYIIISFLVV 1740
MOESM3      FQITTSAGWDGLLAPILNSKPPDCDPKKVHPGSSVEGDCGNPSVGIFYFVSYIIISFLVV 987
            ************************************************************

WT          VNMYIAVILENFSVATEESTEPLSEDDFEMFYEVWEKFDPDATQFIEFSKLSDFAAALDP 1800
MOESM3      VNMYIAVILENFSVATEEST---------------------------------------- 1007
            ********************
```

```
WT          PLLIAKPNKVQLIAMDLPMVSGDRIHCLDILFAFTKRVLGESGEMDSLRSQMEERFMSAN 1860
MOESM3      ------------------------------------------------------------


WT          PSKVSYEPITTTLKRKQEDVSATVIQRAYRRYRLRQNVKNISSIYIKDGDRDDDLLNKKD 1920
MOESM3      ------------------------------------------------------------


WT          MAFDNVNENSSPEKTDATSSTTSPPSYDSVTKPDKEKYEQDRTEKEDKGKDSKESKK 1977
MOESM3      ---------------------------------------------------------
```

2.2 Alignments WT – 6A90

In the following we present the alignments of the WT sequence and the 6A90 sequence. We also present the alignments of their four domains separately.

2.3 Alignment of the whole sequences

The following alignment of the whole sequences has a sequence identity of 31.57%.

```
CLUSTAL O(1.2.4) multiple sequence alignment

WT          -----------------------------------------------MAMLPPPGPQSFV 13
6A90        MASWSHPQFEKGGGARGGSGGGSWSHPQFEKGFDYKDDDDKGTMADNSPLIREERQRLFR 60
                                                             ::     : *

WT          HFTKQSLALIEQRIA-------E--RKSKEPKEEKKDDDEEAPKPSSDLEAGKQLPFIYG 64
6A90        PYTRAMLTAPSAQPAKENGKTEENKDNSRDKGRGANKDRDGSAHPDQALEQGSRLPARMR 120
             :*:  *:  . : *       *   :*::  .  :.* : : :*.. ** *.:**

WT          DI-PPGMVSEPLEDLDPYYADKKTFIVLNKGKTIFRFNATPALYMLSPFSPLRRISIKIL 123
6A90        NIFPAELASTPLEDFDPFYKNKKTFVVVTKAGDIFRFSGEKSLWMLDPFTPIRRVAISTM 180
            :* *  :.* ****:**:* :****:*:.*.  ****..  :*:**.**:*:**::*. :

WT          VHSLFSMLIMCTILTNCIFMTMNNPPDWTKNVEYTFTGIYTFESLVKILARGFCVGEFTF 183
6A90        VQPIFSYFIMITILIHCIFMIMPATQ-TTYILELVFLSIYTIEVVVKVLARGFILHPFAY 239
            *: :** :** *** :**** *      *  :* .* .***:* :**:***** :  *::

WT          LRDPWNWLDFVVIVFAYLTEFVNLGNVSALRTFRVLRALKTISVIPGLKTIVGALIQSVK 243
6A90        LRDPWNWLDFLVTLIGYITLVVDLGHLYALRAFRVLRSWRTVTIVPGWRTIVDALSLSIT 299
            **********:* ::.*:* .*:**:: ***:*****: :*::::** :***.**  *:.

WT          KLSDVMILTVFCLSVFALIGLQLFMGNLKHKCFRNSLENNETLESIMNTLESEEDFRKYF 303
6A90        SLKDLVLLLLFSLFVFAVLGLQIYMGVLTQKCVKHFPADGSWGNFTDERWFNYTSNSSHW 359
            .*.*:::* :*.* ***::***::** *.:**.::   :..  :   :   .  .  .::

WT          YYLEGSKDALLCGFSTDSGQCPEGYTCVK-IGRNPDYGYTSFDTFSWAFLALFRLMTQDY 362
6A90        YIPDDWIEYPLCGNSSGAGMCPPGYTCLQGYGGNPNYGYTSFDTFGWAFLSVFRLVTLDY 419
            *  :.  :  *** *:.:* ** ****::  * **:*********.****::***:* **

WT          WENLYQQTLRAAGKTYMIFFVVVIFLGSFYLINLILAVVAMAYEEQNQANIEEAKQKELE 422
6A90        WEDLYQLALRSAGPWHILFFIIVVFYGTFCFLNFILAVVVMSYTHMVKRADEEKAA-ER- 477
            **:*** :**:**  :::**::*:* *:* ::*:*****.*:* .  :   **    *

WT          FQQMLDRLKKEQEEAEAIAAAAAEYTSIRRSRIMGLSESSSETSKLSSKSAKERRNRRKK 482
6A90        ------ELKKE------------------------------------------------- 482
                  .****

WT          KNQKKLSSGEEKGDAEKLSKSESEDSIRRKSFHLGVEGHRRAHEKRLSTPNQSPLSIRGS 542
6A90        KKAASVANNTANGQE------------------------------------QTTI----- 501
            *:  .::..  :*:                                     *: :
```

```
WT          LFSARRSSRTSLFSFKGRGRDIGSETEFADDEHSIFGDNESRRGSLFVPHRPQERRSSNI 602
6A90        -------------------------EMNGDEAVVIDNNDQAA----------------- 518
                                     *: .**  ::.:*:.

WT          SQASRSPPMLPVNGKMHSAVDCNGVVSLVDGRSALMLPNGQLLPEGTTNQIHKKRRCSSY 662
6A90        ------------------------------------------------------------

WT          LLSEDMLNDPNLRQRAMSRASILTNTVEELEESRQKCPPWWYRFAHKFLIWNCSPYWIKF 722
6A90        ------------R--------------QQSDPETPAPSVTQRLTDFLCVWDCCVPWQKL 551
                        *               : .: .   .*    *::. : :*:*.  * *:

WT          KKCIYFIVMDPFVDLAITICIVLNTLFMAMEHHPMTEEFKNVLAIGNLVFTGIFAAEMVL 782
6A90        QGAIGAVVLSPFFELFIAVIIVLNITFMALDHHDMNIEFERILRTGNYIFTSIYIVEAVL 611
            : .*  :*:.**.:* *:: ****  ***::** *. **:.:*  ** :**.*: .* **

WT          KLIAMDPYEYFQVGWNIFDSLIVTLSLVELFLADVEGLSVLRSFRLLRVFKLAKSWPTLN 842
6A90        KIIALSPKFYFKDSWNVFDFIIVVFAILELGLGEGVQGLSVFRSFRLLRVFRLAKFWPTLN 671
            *:**:.*  **: .**:** :**.::::** * .*:****:*********:*** *****

WT          MLIKIIGNSVGALGNLTLVLAIIVFIFAVVGMQLFGKSYKECVCKINDDCTLPRWHMNDF 902
6A90        NFMSVMTKSYGAFVNVMYVMFLLLFIFAIIGMQLFGMNYIDNMERFP-DGDLPRWNFTDF 730
             ::.:: :* **: *:  *: :::****::****** .* : : ::  *  ****::.**

WT          FHSFLIVFRVLCGEWIETMWDCMEVAGQAMCLIVYMMVMVIGNLVVLNLFLALLLSSFSS 962
6A90        LHSFMIVFRALCGEWIESMWDCMLV-GDWSCIPFFVAVFFVGNLVILNLLIALLLNNYGS 789
            :***:****.*******:***** * *:  *: .:: *:.:****:***::****..:.*

WT          DNLTAIEEDPDANNLQIAVTRIKKGINYVKQTLREFILKAFSKKPKISREIRQAEDLNTK 1022
6A90        FCTSPTSDEEDSKDEDA-LAQI----------VRI--FKRF--KPNL----------N-- 822
               :  .:: *::: :  :::*          :*   :* *  **::          *

WT          KENYISNHTLAEMSKGHNFLKEKDKISGFGSSVDKHLMEDSDGQSFIHNPSLTVTVPIAP 1082
6A90        ---------------------------------------------------AVKLSPMKP 831
                                                               .   *: *

WT          GESDLENMNAEELSSDSDSEYSKVRLNRSSSSECSTVDNPLPGEGEEAEAEPMNSDEPEA 1142
6A90        DSEDIVE--SQ---------------------------EIQGNNIADAEDVLAGEFPPD 861
            ...*: :  ::                             : *:.     : : .: *

WT          CFTDGCVRRFSCC--QVNIESGKGKIWWNIRKTCYKIVEHSWFESFIVLMILLSSGALAF 1200
6A90        CCCN---AFYKCFPSRPARDSSVQRMWSNIRRVCFLLAKNKYFQKFVTAVLVITSVLLAL 918
            *  :     :.*   :   :*.  ::* ***:.*: :.::.:*:.*:. :::::*  **:

WT          EDIYIERKKTIKIILEYADKIFTYIFILEMLLKWIAYGYKTYFTNAWCWLDFLIVDVSLV 1260
6A90        EDIYLPQRPVLVNITLYVDYVLTAFFVIEMIIMLFAVGFKKYFTSKWYWLDFIVVVAYLL 978
            ****: :: .:  *  *.* ::* :*::**::  :* *:*.***. * ****::* . *:

WT          TLVANTLGYSDLGPIKSLRTLRALRPLRALSRFEGMRVVVNALIGAIPSIMNVLLVCLIF 1320
6A90        NFVLMCAGIEA---LQTLRLLRVFRLFRPLSKVNGMQVVTSTLVEAVPHIFNVILVGIFF 1035
            .:*    * .     :::** **.:* :* **:.:**:**..:*: *:* *:**:** ::*

WT          WLIFSIMGVNLFAGKFYECINTTDGSRFPASQVPNRSECFALMNVSQNVRWKNLKVNFDN 1380
6A90        WLVFAIMGVQLFAGKFYKCVDENSTVL-SHEITMDRNDCL-----HENYTWENSPMNFDH 1089
            **:*:****:*******:*:: ..      . . :*.:*:      :*  *:*  :***:

WT          VGLGYLSLLQVATFKGWTIIMYAAVDSVNVDKQPKYEYSLYMYIYFVVFIIFGSFFTLNL 1440
6A90        VGNAYLSLLQVATFKGWLQIMNDAIDSREVHKQPIRETNIYMYLYFIFFIVFGSFFILKL 1149
            ** .*************  **  *:** :*.***  * .:***:**:.**:***** *:*

WT          FIGVIIDNFNQQKKKLGGQDIFMTEEQKKYYNAMKKLGSKKPQKPIPRPGNKIQGCIFDL 1500
6A90        FVCILIDIFRQQRRKAEGLSATDSRTQLIYRRAVMRTMSAKPVKRIPKPTCHPQSLMYDI 1209
            *: ::** *.**::*  * .    :. *  * .*: :  * ** * **:*  : *. ::*:

WT          VTNQAFDISIMVLICLNMVTMMVEKEGQSQHMTEVLYWINVVFIILFTGECVLKLISLRH 1560
6A90        SVNRKFEYTMMILIILNVAVMAIDHYGQSMEFSEVLDYLNLIFIIIFFVECVIKVSGLRH 1269
             .*: *: ::*:** **:..* ::: *** .::*** ::*::***:*  ***:*: .***
```

```
WT           YYFTVGWNIFDFVVVIISIVGMFLADLIETYFVSPTLFRVIRLARIGRILRLVKGAKGIR 1620
6A90         HYFKDPWNIIDFLYVVLAIAGLMLSDVIEKYFISPTLLRILRILRVGRLLRYFQSARGMR 1329
             :**.  ***:**: *:::*.*::*:*:**.**:****:*::*: *:**:** .:.*:*:*

WT           TLLFALMMSLPALFNIGLLLFLVMFIYAIFGMSNFAYVKKEDGINDMFNFETFGNSMICL 1680
6A90         LLLLALRKALRTLFNVSFLLFVIMFVYAVFGMEFFMHIRDAGAIDDVYNFKTFGQSIILL 1389
              **:**  :* :***:.:***::**:**:***. * :::. ..*:*::**:***:*:* *

WT           FQITTSAGWDGLLAPILNSKPPDCDPKKVHPGSSVEGDCGNPSVGIFYFVSYIIISFLVV 1740
6A90         FQLATSAGWDGVYFAIANEE--DCRAPD--HELGYPGNCGSRALGIAYLVSYLIITCLVV 1445
             **::*******:   * *.:  **   .     .  *:**. ::** *:***:**: ***

WT           VNMYIAVILENFSVATEESTEPLSEDDFEMFYEVWEKFDPDATQFIEFSKLSDFAAALDP 1800
6A90         INMYAAVILDYVLEVYEDSKEGLTDDDYDMFFEVWQQFDPEATQYIRYDQLSELLEALQP 1505
             :*** ****: .  . *:*.* *::**::**:***::***:***:*.:.:**::  **:*

WT           PLLIAKPNKVQLIAMDLPMVSGDRIHCLDILFAFTKRVLGESGEMDSLRSQMEERFMSAN 1860
6A90         PLQVQKPNKYKILSMNIPICKDDHIFYKDVLEALVKDVFSRRGSPVEA-----GDVQAPN 1560
             ** : **** ::::*::*: ..*:*.  *:* *:.* *:.. *.  .        . : *

WT           PSKVSYEPITTTLKRKQEDVSATVIQRAYRRYRLRQNVKNISSIYIKDGDRDDDLLNKKD 1920
6A90         VDEAEYKPVSSTLQRQREEYCVRLIQNAWRKHKQQN------------------------ 1596
              .:..*:*::**:*::*: .. :**.*:*::: ::

WT           MAFDNVNENSSPEKTDATSSTTSPPSYDSVTKPDKEKYEQDRTEKEDKGKDSKESKK 1977
6A90         ---------------------------------------------------------
```

2.3.1 Alignment of the first domain DI

For the first domain the sequence identity is 43%.

```
CLUSTAL O(1.2.4) multiple sequence alignment

WT - DI        FSPLRRISIKILVHSLFSMLIMCTILTNCIFMTMNNPPDWTKNVEYTFTGIYTFESLVKI 60
6A90 - DI      ---------------IFSYFIMITILIHCIFMIMPATQ-TTYILELVFLSIYTIEVVVKV 44
                              :** :** *** :**** *      *  :* .* .***:* :**:

WT - DI        LARGFCVGEFTFLRDPWNWLDFVVIVFAYLTEFVNLGNVSALRTFRVLRALKTISVIPGL 120
6A90 - DI      LARGFILHPFAYLRDPWNWLDFLVTLIGYITLVVDLGHLYALRAFRVLRSWRTVTIVPGW 104
               ***** :  *::**********:* ::.*:* .*:**:: ***:*****: :*::::**

WT - DI        KTIVGALIQSVKKLSDVMILTVFCLSVFALIGLQLFMGNLKHKCFRNSLENNETLESIMN 180
6A90 - DI      RTIVDALSLSITSLKDLVLLLLFSLFVFAVLGLQIYMGVLTQKCVKHFPADGSWGNFTDE 164
               :***.**  *:..*.*:::* :*.* ***::***::** *.:**.::   :..  :   :

WT - DI        TLESEEDFRKYFYYLEGSKDALLCGFSTDSGQCPEGYTCVK-IGRNPDYGYTSFDTFSWA 239
6A90 - DI      RWFNYTSNSSHWYIPDDWIEYPLCGNSSGAGMCPPGYTCLQGYGGNPNYGYTSFDTFGWA 224
                  .  .  .::*  :.  :  *** *:.:* ** ****::  * **:*********.**

WT - DI        FLALFRLMTQDYWENLYQQTLRAAGKTYMIFFVVVIFLGSFYLINLILAVVAMAYEEQNQ 299
6A90 - DI      FLSVFRLVTLDYWEDLYQLALRSAGPWHILFFIIVVFYGTFCFLNFILA----------- 273
               **::***:* ****:*** :**:**  ::**::*:* *:* ::*:***
```

2.3.2 Alignment of the second domain DII

For the second domain the sequence identity is 43.561%.

```
CLUSTAL O(1.2.4) multiple sequence alignment

WT - DII         CSPYWIKFKKCIYFIVMDPFVDLAITICIVLNTLFMAMEHHPMTEEFKNVLAIGNLVFTG 60
DII-6A90         -------------------FFELFIAVIIVLNITFMALDHHDMNIEFERILRTGNYIFTS 41
                                    *.:* *:: ****  ***::** *. **:.:*  ** :**.
```

```
WT - DII        IFAAEMVLKLIAMDPYEYFQVGWNIFDSLIVTLSLVELFLADVEGLSVLRSFRLLRVFKL 120
DII-6A90        IYIVEAVLKIIALSPKFYFKDSWNVFDFIIVVFAILELGLEG--VLSVFRSFRLLRVFRL 99
                *: .* ***:**:.*  **: .**:** :**.::::** * .   ***:*********:*

WT - DII        AKSWPTLNMLIKIIGNSVGALGNLTLVLAIIVFIFAVVGMQLFGKSYKECVCKINDDCTL 180
DII-6A90        AKFWPTLNNFMSVMTKSYGAFVNVMYVMFLLLFIFAIIGMQLFGMNYIDNMERFP-DGDL 158
                ** ***** ::.:: :* **: *:  *: :::****::****** .* : : ::  *  *

WT - DII        PRWHMNDFFHSFLIVFRVLCGEWIETMWDCMEVAGQAMCLIVYMMVMVIGNLVVLNLFLA 240
DII-6A90        PRWNFTDFLHSFMIVFRALCGEWIESMWDCMLV-GDWSCIPFFVAVFFVGNLVILNLLIA 217
                ***::.**:***:****.*******:***** * *:  *: .:: *:.:****:***::*

WT - DII        LLLSSFSSDNLTAIEEDPDANNLQ 264
DII-6A90        LL---------------------- 219
                **
```

2.3.3 Alignment of the third domain DIII

For the third domain the sequence identity is 37.217%.

```
CLUSTAL O(1.2.4) multiple sequence alignment

WT - DIII        NIRKTCYKIVEHSWFESFIVLMILLSSGALAFEDIYIERKKTIKIILEYADKIFTYIFIL 60
6A90 - DIII      --------------FQKFVTAVLVITSVLLALEDIYLPQRPVLVNITLYVDYVLTAFFVI 46
                               *:.*:. :::::*  **:****: :: .:  *  *.* ::* :*::

WT - DIII        EMLLKWIAYGYKTYFTNAWCWLDFLIVDVSLVTLVANTLGYSDLGPIKSLRTLRALRPLR 120
6A90 - DIII      EMIIMLFAVGFKKYFTSKWYWLDFIVVVAYLLNFVLMCAGIE---ALQTLRLLRVFRLFR 103
                 **::  :* *:*.***. * ****::* . *:.:*    * .    :::** **.:* :*

WT - DIII        ALSRFEGMRVVVNALIGAIPSIMNVLLVCLIFWLIFSIMGVNLFAGKFYECINTTDGSRF 180
6A90 - DIII      PLSKVNGMQVVTSTLVEAVPHIFNVILVGIFFWLVFAIMGVQLFAGKFYKCVDENSTV-L 162
                  **:.:**:**..:*: *:* *:**:** ::***:*:****:*******:*:: ..   :

WT - DIII        PASQVPNRSECFALMNVSQNVRWKNLKVNFDNVGLGYLSLLQVATFKGWTIIMYAAVDSV 240
6A90 - DIII      SHEITMDRNDCL-----HENYTWENSPMNFDHVGNAYLSLLQVATFKGWLQIMNDAIDSR 217
                   . . :*.:*:      :*  *:*  :***:** .*************  **  *:**

WT - DIII        NVDKQPKYEYSLYMYIYFVVFIIFGSFFTLNLFIGVIIDNFNQQKKKLGGQDIFMTEEQK 300
6A90 - DIII      EVHKQPIRETNIYMYLYFIFFIVFGSFFILKLFV-------------------------- 251
                 :*.***  * .:***:**:.**:***** *:**:

WT - DIII        KYYNAMKKL 309
6A90 - DIII      ---------
```

2.3.4 Alignment of the fourth domain DIV

For the fourth domain the sequence identity is 39.13%.

```
CLUSTAL O(1.2.4) multiple sequence alignment

WT - DIV        IPRPGNKIQGCIFDLVTNQAFDISIMVLICLNMVTMMVEKEGQSQHMTEVLYWINVVFII 60
6A90 - DIV      --------------------FEYTMMILIIILNVAVMAIDHYGQSMEFSEVLDYLNLIFII 40
                                    *: ::*:** **:..* ::: *** .::*** ::*::***

WT - DIV        LFTGECVLKLISLRHYYFTVGWNIFDFVVVIISIVGMFLADLIETYFVSPTLFRVIRLAR 120
6A90 - DIV      IFFVECVIKVSGLRHHYFKDPWNIIDFLYVVLAIAGLMLSDVIEKYFISPTLLRILRILR 100
                :*  ***:*: .***:**.  ***:**: *:::*.*::*:*:**.**:****:*::*: *

WT - DIV        IGRILRLVKGAKGIRTLLFALMMSLPALFNIGLLLFLVMFIYAIFGMSNFAYVKKEDGIN 180
6A90 - DIV      VGRLLRYFQSARGMRLLLLALRKALRTLFNVSFLLFVIMFVYAVFGMEFFMHIRDAGAID 160
                :**:** .:.*:*:* **:**  :* :***:.:***::**:**:***. * :::. ..*:
```

```
WT - DIV        DMFNFETFGNSMICLFQITTSAGWDGLLAPILNSKPPDCDPKKVHPGSSVEGDCGNPSVG 240
6A90 - DIV      DVYNFKTFGQSIILLFQLATSAGWDGVYFAIANEE--DCRAPD--HELGYPGNCGSRALG 216
                *::**:***:*:* ***::*******:   * *.:  **   .     .  *:**. ::*

WT - DIV        IFYFVSYIIISFLVVVNMYIAVILENFSVATEESTEPLSEDDFEMFYEVWEKFDPDATQ 299
6A90 - DIV      IAYLVSYLIITCLVVINMYAAVI------------------------------------ 239
                * *:***:**: ***:*** ***
```

2.4 Alignment WT – 6J8J

We present the alignment between the whole sequences of the WT and the 6J8J template: their sequence identity is the 97.243%.

```
CLUSTAL O(1.2.4) multiple sequence alignment

WT          -------------------------------------------MAMLPPPGPQSFVHFTK 17
6J8J        MASWSHPQFEKGGGARGGSGGGSWSHPQFEKGFDYKDDDDKGTMAMLPPPGPQSFVHFTK 60
                                                       *****************

WT          QSLALIEQRIAERKSKEPKEEKKDDDEEAPKPSSDLEAGKQLPFIYGDIPPGMVSEPLED 77
6J8J        QSLALIEQRIAERKSKEPKEEKKDDDEEAPKPSSDLEAGKQLPFIYGDIPPGMVSEPLED 120
            ************************************************************

WT          LDPYYADKKTFIVLNKGKTIFRFNATPALYMLSPFSPLRRISIKILVHSLFSMLIMCTIL 137
6J8J        LDPYYADKKTFIVLNKGKTIFRFNATPALYMLSPFSPLRRISIKILVHSLFSMLIMCTIL 180
            ************************************************************

WT          TNCIFMTMNNPPDWTKNVEYTFTGIYTFESLVKILARGFCVGEFTFLRDPWNWLDFVVIV 197
6J8J        TNCIFMTMNNPPDWTKNVEYTFTGIYTFESLVKILARGFCVGEFTFLRDPWNWLDFVVIV 240
            ************************************************************

WT          FAYLTEFVNLGNVSALRTFRVLRALKTISVIPGLKTIVGALIQSVKKLSDVMILTVFCLS 257
6J8J        FAYLTEFVNLGNVSALRTFRVLRALKTISVIPGLKTIVGALIQSVKKLSDVMILTVFCLS 300
            ************************************************************

WT          VFALIGLQLFMGNLKHKCFRNSLENNETLESIMNTLESEEDFRKYFYYLEGSKDALLCGF 317
6J8J        VFALIGLQLFMGNLKHKCFRNSLENNETLESIMNTLESEEDFRKYFYYLEGSKDALLCGF 360
            ************************************************************

WT          STDSGQCPEGYTCVKIGRNPDYGYTSFDTFSWAFLALFRLMTQDYWENLYQQTLRAAGKT 377
6J8J        STDSGQCPEGYTCVKIGRNPDYGYTSFDTFSWAFLALFRLMTQDYWENLYQQTLRAAGKT 420
            ************************************************************

WT          YMIFFVVVIFLGSFYLINLILAVVAMAYEEQNQANIEEAKQKELEFQQMLDRLKKEQEEA 437
6J8J        YMIFFVVVIFLGSFYLINLILAVVAMAYKEQNQANIEEAKQKELEFQQMLDRLKKEQEEA 480
            ****************************:*******************************

WT          EAIAAAAAEYTSIRRSRIMGLSESSSETSKLSSKSAKERRNRRKKKNQKKLSSGEEKGDA 497
6J8J        EAIAAAAAEYTSIRRSRIMGLSESSSETSKLSSKSAKERRNRRKKKNQKKLSSGEEKGDA 540
            ************************************************************

WT          EKLSKSESEDSIRRKSFHLGVEGHRRAHEKRLSTPNQSPLSIRGSLFSARRSSRTSLFSF 557
6J8J        EKLSKSESEDSIRRKSFHLGVEGHRRAHEKRLSTPNQSPLSIRGSLFSARRSSRTSLFSF 600
            ************************************************************

WT          KGRGRDIGSETEFADDEHSIFGDNESRRGSLFVPHRPQERRSSNISQASRSPPMLPVNGK 617
6J8J        KGRGRDIGSETEFADDEHSIFGDNESRRGSLFVPHRPQERRSSNISQASRSPPMLPVNGK 660
            ************************************************************

WT          MHSAVDCNGVVSLVDGRSALMLPNGQLLPE-----------GTTNQIHKKRRCSSYLLSE 666
6J8J        MHSAVDCNGVVSLVDGRSALMLPNGQLLPEVIIDKATSDDSGTTNQIHKKRRCSSYLLSE 720
            ******************************           *******************

WT          DMLNDPNLRQRAMSRASILTNTVEELEESRQKCPPWWYRFAHKFLIWNCSPYWIKFKKCI 726
6J8J        DMLNDPNLRQRAMSRASILTNTVEELEESRQKCPPWWYRFAHKFLIWNCSPYWIKFKKCI 780
            ************************************************************
```

```
WT          YFIVMDPFVDLAITICIVLNTLFMAMEHHPMTEEFKNVLAIGNLVFTGIFAAEMVLKLIA 786
6J8J        YFIVMDPFVDLAITICIVLNTLFMAMEHHPMTEEFKNVLAIGNLVFTGIFAAEMVLKLIA 840
            ************************************************************

WT          MDPYEYFQVGWNIFDSLIVTLSLVELFLADVEGLSVLRSFRLLRVFKLAKSWPTLNMLIK 846
6J8J        MDPYEYFQVGWNIFDSLIVTLSLVELFLADVEGLSVLRSFRLLRVFKLAKSWPTLNMLIK 900
            ************************************************************

WT          IIGNSVGALGNLTLVLAIIVFIFAVVGMQLFGKSYKECVCKINDDCTLPRWHMNDFFHSF 906
6J8J        IIGNSVGALGNLTLVLAIIVFIFAVVGMQLFGKSYKECVCKINDDCTLPRWHMNDFFHSF 960
            ************************************************************

WT          LIVFRVLCGEWIETMWDCMEVAGQAMCLIVYMMVMVIGNLVVLNLFLALLLSSFSSDNLT 966
6J8J        LIVFRVLCGEWIETMWDCMEVAGQAMCLIVYMMVMVIGNLVVLNLFLALLLSSFSSDNLT 1020
            ************************************************************

WT          AIEEDPDANNLQIAVTRIKKGINYVKQTLREFILKAFSKKPKISREIRQAEDLNTKKENY 1026
6J8J        AIEEDPDANNLQIAVTRIKKGINYVKQTLREFILKAFSKKPKISREIRQAEDLNTKKENY 1080
            ************************************************************

WT          ISNHTLAEMSKGHNFLKEKDKISGFGSSVDKHLMEDSDGQSFIHNPSLTVTVPIAPGESD 1086
6J8J        ISNHTLAEMSKGHNFLKEKDKISGFGSSVDKHLMEDSDGQSFIHNPSLTVTVPIAPGESD 1140
            ************************************************************

WT          LENMNAEELSSDSDSEYSKVRLNRSSSSECSTVDNPLPGEGEEAEAEPMNSDEPEACFTD 1146
6J8J        LENMNAEELSSDSDSEYSKVRLNRSSSSECSTVDNPLPGEGEEAEAEPMNSDEPEACFTD 1200
            ************************************************************

WT          GCVRRFSCCQVNIESGKGKIWWNIRKTCYKIVEHSWFESFIVLMILLSSGALAFEDIYIE 1206
6J8J        GCVWRFSCCQVNIESGKGKIWWNIRKTCYKIVEHSWFESFIVLMILLSSGALAFEDIYIE 1260
            *** ********************************************************

WT          RKKTIKIILEYADKIFTYIFILEMLLKWIAYGYKTYFTNAWCWLDFLIVDVSLVTLVANT 1266
6J8J        RKKTIKIILEYADKIFTYIFILEMLLKWIAYGYKTYFTNAWCWLDFLIVDVSLVTLVANT 1320
            ************************************************************

WT          LGYSDLGPIKSLRTLRALRPLRALSRFEGMRVVVNALIGAIPSIMNVLLVCLIFWLIFSI 1326
6J8J        LGYSDLGPIKSLRTLRALRPLRALSRFEGMRVVVNALIGAIPSIMNVLLVCLIFWLIFSI 1380
            ************************************************************

WT          MGVNLFAGKFYECINTTDGSRFPASQVPNRSECFALMNVSQNVRWKNLKVNFDNVGLGYL 1386
6J8J        MGVNLFAGKFYECINTTDGSRFPASQVPNRSECFALMNVSQNVRWKNLKVNFDNVGLGYL 1440
            ************************************************************

WT          SLLQVATFKGWTIIMYAAVDSVNVDKQPKYEYSLYMYIYFVVFIIFGSFFTLNLFIGVII 1446
6J8J        SLLQVATFKGWTIIMYAAVDSVNVDKQPKYEYSLYMYIYFVVFIIFGSFFTLNLFIGVII 1500
            ************************************************************

WT          DNFNQQKKKLGGQDIFMTEEQKKYYNAMKKLGSKKPQKPIPRPGNKIQGCIFDLVTNQAF 1506
6J8J        DNFNQQKKKLGGQDIFMTEEQKKYYNAMKKLGSKKPQKPIPRPGNKIQGCIFDLVTNQAF 1560
            ************************************************************

WT          DISIMVLICLNMVTMMVEKEGQSQHMTEVLYWINVVFIILFTGECVLKLISLRHYYFTVG 1566
6J8J        DISIMVLICLNMVTMMVEKEGQSQHMTEVLYWINVVFIILFTGECVLKLISLRHYYFTVG 1620
            ************************************************************

WT          WNIFDFVVVIISIVGMFLADLIETYFVSPTLFRVIRLARIGRILRLVKGAKGIRTLLFAL 1626
6J8J        WNIFDFVVVIISIVGMFLADLIETYFVSPTLFRVIRLARIGRILRLVKGAKGIRTLLFAL 1680
            ************************************************************

WT          MMSLPALFNIGLLLFLVMFIYAIFGMSNFAYVKKEDGINDMFNFETFGNSMICLFQITTS 1686
6J8J        MMSLPALFNIGLLLFLVMFIYAIFGMSNFAYVKKEDGINDMFNFETFGNSMICLFQITTS 1740
            ************************************************************

WT          AGWDGLLAPILNSKPPDCDPKKVHPGSSVEGDCGNPSVGIFYFVSYIIISFLVVVNMYIA 1746
6J8J        AGWDGLLAPILNSKPPDCDPKKVHPGSSVEGDCGNPSVGIFYFVSYIIISFLVVVNMYIA 1800
            ************************************************************
```

```
WT          VILENFSVATEESTEPLSEDDFEMFYEVWEKFDPDATQFIEFSKLSDFAAALDPPLLIAK 1806
6J8J        VILENFSVATEESTEPLSEDDFEMFYEVWEKFDPDATQFIEFSKLSDFAAALDPPLLIAK 1860
            ************************************************************

WT          PNKVQLIAMDLPMVSGDRIHCLDILFAFTKRVLGESGEMDSLRSQMEERFMSANPSKVSY 1866
6J8J        PNKVQLIAMDLPMVSGDRIHCLDILFAFTKRVLGESGEMDSLRSQMEERFMSANPSKVSY 1920
            ************************************************************

WT          EPITTTLKRKQEDVSATVIQRAYRRYRLRQNVKNISSIYIKDGDRDDDLLNKKDMAFDNV 1926
6J8J        EPITTTLKRKQEDVSATVIQRAYRRYRLRQNVKNISSIYIKDGDRDDDLLNKKDMAFDNV 1980
            ************************************************************

WT          NENSSPEKTDATSSTTSPPSYDSVTKPDKEKYEQDRTEKEDKGKDSKESKK 1977
6J8J        NENSSPEKTDATSSTTSPPSYDSVTKPDKEKYEQDRTEKEDKGKDSKESKK 2031
            ***************************************************
```

3 Quality assessment: detailed analysis

In this section we report further results concerning the quality assessment of the three-dimensional structures obtained with our computational pipeline.

Each structure obtained by FG-MD was subjected to an quality evaluation with the tool QMEANBrane, which has a special scoring function designed for membrane proteins. As reported in the paper, QMEANBrane shows that the produced models are of high quality within each domain area, while, in the inter-domains loops area, the reliability of the models are significantly lower.

Figure S1 shows the quality results for the MOESM3 template. In particular, part (a) depicts the structure of the WT and part (b) shows the quality values along peptide sequence: the higher values correspond to positions falling into the transmembrane region. Moreover, part (c) of Figure S1 shows the RAMPAGE results for the MOESM3 WT. The analysis considers only the protein segments corresponding to the transmembrane region, i.e. all the α-helices. It turns out that 95.8% amino acids fall within a favorable region, 4% fall within a permitted region and only one amino acid falls in the forbidden region.

The same analysis is reported in Figure S2 for template 6J8J: it shows that the percentage of amino acids in the favorable zone is 98.7%, while amino acids in the permitted area are 0.9% of the whole modeled sequence.

To complete the quality assessment, the local quality values of all the considered point mutations are reported in Tables S3 and S4 for the MOESM3 template; in Tables S5 and S6 for the 6A90 template and in Tables S7 and S8 for the 6J8J template.

A further quality check was performed on the Ramachandran plot of the WT structure of the three considered templates: for each genetic variant of the two groups **PAT** and **NEUTRAL**, we check if the corresponding amino acid is present in a high quality portion of the protein reported by QMEANBrane. The results are reported in Tables S9 and S10.

**QMEANBrane results: MOESM3 - PAT** mutation			
	Mutation	WT	Mutated
**IEM**	I136V	0.86	0.9
	S211P	0.73	0.8
	F216S	0.8	0.82
	I234T	0.81	0.84
	S241T	0.88	0.92
	N395K	0.85	0.83
	V400M	0.92	0.94
	L823R	0.82	0.78
	I848T	0.79	0.79
	L858H	0.8	0.77
	L859F	0.8	0.61
	A863P	0.9	0.87
	V872G	0.87	0.88
	P1308L	0.61	0.74
	V1316A	0.85	0.88
	F1449V	0.84	0.89
	W1538R	0.6	0.57
	A1746G	0.91	0.91
**PEPD**	V1298D	0.77	0.87
	V1298F	0.77	0.72
	V1299F	0.77	0.81
	G1607R	0.83	0.76
	M1627K	0.77	0.74
	A1632E	0.82	0.77
**SFN**	R185H	0.29	0.38
	I228M	0.77	0.77
	I739V	0.84	0.87
	G856D	0.72	0.71
	M932L	0.76	0.73
	M1532I	0.62	0.49
**PDN**	T1596I	0.84	0.75

Table S3: QMEANBrane results for the models generated by the MOESM3 template: quality value of each point mutation in the **PAT** group before (WT) and after (mutated) the amino acid change.

4 Energy Landscape

A pivotal step, immediately following homology modeling, is the step involving the energy minimization. It is well known the connection between function and structure of a protein, therefore mutations that cause similar physiological changes will also have similar structures. We used the same starting geometry (reference template) for all the protein sequences that we modeled. And the basic idea is that the energy minimization step, by taking into account the punctual amino acid differences, would have led the models to divide into two groups characterized by similar geometries. Indeed, these two groups of similar geometries correspond precisely to the two groups having different physiological behaviors (see Figure S3).

**QMEANBrane results: MOESM3 - NEUTRAL** variant					
Mut	WT(FG-MD)	Mut(FG-MD)	Mut	WT(FG-MD)	Mut(FG-MD)
S126A	0.81	0.77	A1505V	0.73	0.72
L127A	0.81	0.84	S1509A	0.9	0.93
M145L	0.54	0.77	S1509T	0.9	0.87
N146S	0.53	0.73	Q1530D	0.69	0.56
V194I	0.85	0.84	Q1530K	0.69	0.42
L201V	0.8	0.82	Q1530P	0.69	0.62
N206D	0.73	0.6	H531Y	0.7	0.47
T370M	0.64	0.56	M1532V	0.62	0.62
E759D	0.65	0.74	E1534D	0.47	0.58
A766T	0.76	0.78	Y1537N	0.55	0.64
A766V	0.76	0.81	T1548S	0.88	0.89
I767V	0.76	0.85	H1560C	0.67	0.77
T773S	0.82	0.83	H1560Y	0.67	0.58
V795I	0.84	0.77	V1565I	0.8	0.71
A815S	0.76	0.88	I1577L	0.81	0.85
D890E	0.54	0.51	D1586E	0.75	0.6
D890V	0.54	0.51	T1590K	0.5	0.53
T920N	0.64	0.73	T1590R	0.5	0.58
K1176R	0.32	0.54	V1613I	0.78	0.78
R1207K	0.66	0.68	D1662A	0.52	0.58
T1210N	0.75	0.77	G1674A	0.84	0.84
I1235V	0.71	0.86	K1700A	0.63	0.67
N1245S	0.72	0.74			
L1267V	0.79	0.89			
T1398M	0.77	0.7			
I1399D	0.74	0.79			
D1411N	0.55	0.55			
K1412E	0.55	0.62			
K1412I	0.55	0.67			
K1415I	0.61	0.67			
S1419N	0.84	0.84			
V1428I	0.88	0.86			

Table S4: QMEANBrane results for the models generated by the MOESM3 template: quality value of each point mutation in the **NEUTRAL** group before (WT) and after (mutated) the amino acid change.

**QMEANBrane results: 6A90 - PAT** mutations			
	Mut	WT(FG-MD)	Mut(FG-MD)
**IEM**	I136V	0.85	0.89
	S211P	0.68	0.78
	F216S	0.78	0.83
	I234T	0.86	0.86
	S241T	0.93	0.94
	N395K	0.86	0.88
	V400M	0.99	0.94
	L823R	0.74	0.74
	I848T	0.78	0.75
	L858H	0.88	0.82
	L859F	0.88	0.89
	A863P	0.98	0.97
	V872G	0.86	0.88
	P1308L	0.8	0.76
	V1316A	0.9	0.93
	F1449V	0.95	0.89
	W1538R	0.96	1.0
	A1746G	0.96	0.99
**PEPD**	V1298D	0.77	0.87
	V1298F	0.92	0.89
	V1299F	0.92	0.93
	G1607R	0.85	0.76
	M1627K	0.88	0.9
	A1632E	0.9	0.84
**SFN**	R185H	0.46	0.6
	I228M	0.72	0.72
	I739V	0.92	0.96
	G856D	0.86	0.8
	M932L	0.77	0.79
	M1532I	0.84	0.79
**PDN**	T1596I	0.8	0.81

Table S5: QMEANBrane results for the models generated by the 6A90 template: quality value of each point mutation in the **PAT** group before (WT) and after (mutated) the amino acid change.

**QMEANBrane results: 6A90 - NEUTRAL** variant					
Mut	WT(FG-MD)	Mut(FG-MD)	Mut	WT(FG-MD)	Mut(FG-MD)
S126A	0.72	0.77	A1505V	0.87	0.82
L127A	0.78	0.77	S1509A	0.93	0.89
M145L	0.73	0.77	S1509T	0.93	0.97
N146S	0.79	0.69	Q1530D	0.87	0.67
V194I	0.91	0.88	Q1530K	0.87	0.73
L201V	0.69	0.75	Q1530P	0.87	0.77
N206D	0.47	0.32	H531Y	0.88	1.0
T370M	0.93	0.92	M1532V	0.84	0.88
E759D	0.87	0.9	E1534D	0.88	0.88
A766T	0.88	0.92	Y1537N	0.91	0.91
A766V	0.88	0.92	T1548S	0.93	0.94
I767V	0.84	0.93	H1560C	0.64	0.65
T773S	0.9	0.93	H1560Y	0.64	0.7
V795I	0.75	0.69	V1565I	0.72	0.72
A815S	0.66	0.74	I1577L	0.86	0.9
D890E	0.53	0.59	D1586E	0.58	0.42
D890V	0.53	0.49	T1590K	0.47	0.38
T920N	0.92	0.89	T1590R	0.47	0.42
K1176R	0.74	0.64	V1613I	0.77	0.78
R1207K	0.59	0.71	D1662A	0.7	0.63
T1210N	0.82	0.69	G1674A	0.87	0.85
I1235V	0.84	0.83	K1700A	0.64	0.68
N1245S	0.74	0.76			
L1267V	0.34	0.05			
T1398M	0.9	0.9			
I1399D	0.86	0.86			
D1411N	0.67	0.7			
K1412E	0.68	0.65			
K1412I	0.68	0.63			
K1415I	0.66	0.66			
S1419N	0.76	0.75			
V1428I	0.88	0.86			

Table S6: QMEANBrane results for the models generated by the 6A90 template: quality value of each point mutation in the **NEUTRAL** group before (WT) and after (mutated) the amino acid change.

**QMEANBrane results: 6J8J - PAT** mutations			
	Mut	WT(FG-MD)	Mut(FG-MD)
**IEM**	I136V	0.85	0.90
	S211P	0.82	0.88
	F216S	0.80	0.81
	I234T	0.80	0.91
	S241T	0.89	0.93
	N395K	0.92	0.89
	V400M	0.95	0.93
	L823R	0.77	0.70
	I848T	0.85	0.82
	L858H	0.84	0.78
	L859F	0.84	0.85
	A863P	0.97	0.98
	V872G	0.90	0.91
	P1308L	0.78	0.81
	V1316A	0.92	0.92
	F1449V	0.85	0.92
	W1538R	1.00	1.0
	A1746G	0.95	0.97
**PEPD**	V1298D	0.82	0.81
	V1298F	0.82	0.86
	V1299F	0.85	0.87
	G1607R	0.92	0.82
	M1627K	0.90	0.87
	A1632E	0.90	0.88
**SFN**	R185H	0.46	0.65
	I228M	0.75	0.73
	I739V	0.88	0.93
	G856D	0.79	0.73
	M932L	0.93	0.90
	M1532I	0.87	0.88
**PDN**	T1596I	0.79	0.78

Table S7: QMEANBrane results for the models generated by the 6J8J template: quality value of each point mutation in the **PAT** group before (WT) and after (mutated) the amino acid change.

**QMEANBrane results: 6J8J - NEUTRAL** variants					
Mut	WT(FG-MD)	Mut(FG-MD)	Mut	WT(FG-MD)	Mut(FG-MD)
S126A	0.80	0.86	A1505V	0.87	0.84
L127A	0.80	0.84	S1509A	0.91	0.95
M145L	0.70	0.75	S1509T	0.91	0.91
N146S	0.73	0.85	Q1530D	0.84	0.96
V194I	0.87	0.86	Q1530K	0.84	0.93
L201V	0.70	0.77	Q1530P	0.84	0.78
N206D	0.52	0.66	H531Y	0.89	1.0
T370M	0.94	0.94	M1532V	0.87	0.97
E759D	0.72	1.00	E1534D	0.90	0.76
A766T	0.90	0.86	Y1537N	0.98	0.90
A766V	0.90	0.91	T1548S	0.96	0.98
I767V	0.85	0.90	H1560C	0.77	0.72
T773S	0.90	0.92	H1560Y	0.77	0.54
V795I	0.76	0.70	V1565I	0.77	0.70
A815S	0.75	0.77	I1577L	0.91	0.94
D890E	0.72	0.69	D1586E	0.74	0.78
D890V	0.72	0.64	T1590K	0.73	0.76
T920N	0.93	0.91	T1590R	0.73	0.82
K1176R	0.82	0.68	V1613I	0.80	0.80
R1207K	0.59	0.70	D1662A	0.68	0.67
T1210N	0.83	0.80	G1674A	0.88	0.88
I1235V	0.87	0.92	K1700A	0.66	0.64
N1245S	0.59	0.74			
L1267V	0.74	0.74			
T1398M	0.92	0.88			
I1399D	0.88	0.87			
D1411N	0.66	0.67			
K1412E	0.68	0.71			
K1412I	0.68	0.64			
K1415I	0.66	0.62			
S1419N	0.75	0.64			
V1428I	0.93	0.92			

Table S8: QMEANBrane results for the models generated by the 6J8J template: quality value of each point mutation in the **NEUTRAL** group before (WT) and after (mutated) the amino acid change.

Ramachandran Plot Analysis of WT structures and PAT mutations				
	Mutation	MOESM3(FG-MD)	6A90(FG-MD)	6J8J(FG-MD)
**IEM**	I136	√	√	√
	S211	√	×○	√
	F216	√	√	√
	I234	√	√	√
	S241	√	√	√
	N395	√	√	√
	V400	√	√	√
	L823	√	√	√
	I848	√	√	√
	L858	√	√	√
	A863	√	√	√
	V872	√	√	√
	P1308	√	√	√
	V1316	√	√	√
	F1449	√	√	√
	W1538	√	√	√
	A1746	√	√	√
**PEPD**	V1298	√	√	√
	V1299	√	√	√
	G1607	√	√	√
	M1627	√	√	√
	A1632	√	√	√
**SFN**	R185	×	√	×○
	I228	×○	×○	×○
	I739	√	√	√
	G856	√	√	√
	M932	√	√	√
	M1532	√	√	√
**PDN**	T1596	√	√	√

Table S9: Presence/Absence of the **PAT** mutations on the Ramachandran plot of the WT structures of the three considered templates. Only high quality parts of the models have been considered for the RAMPAGE analysis. Legend: √= residue present; ×=residue not present and ×○= residue not present, but its first neighbor yes.

**Ramachandran Plot Analysis of WT structures and NEUTRAL genetic variants**							
Mutation	M3(FG-MD)	6A(FG-MD)	6J(FG-MD)	Mutation	M3(FG-MD)	6A(FG-MD)	6J(FG-MD)
S126	√	√	√	A1505	√	√	√
L127	√	√	√	S1509	√	√	√
M145	×◦	×◦	√	Q1530	√	√	√
N146	×	×	×◦	H1531	√	√	√
V194	√	√	√	M1532	√	√	√
L201	√	√	√	E1534	×◦	√	√
N206	×	×	×	Y1537	√	√	√
T370	×	√	√	T1548	√	√	√
E759	√	√	√	H1560	√	×	√
A766	√	√	√	V1565	×◦	×◦	√
I767	√	√	√	I1577	√	√	√
T773	√	√	√	D1586	√	×	√
V795	×◦	×◦	×◦	T1590	×	×	√
A815	×◦	×	√	V1613	√	×◦	√
D890	×	×	×	D1662	√	×	×
T920	×	√	√	G1674	√	√	√
K1176	×	√	√	K1700	×	×	×
R1207	√	×	×				
T1210	√	√	√				
I1235	√	√	√				
N1245	×◦	×◦	×◦				
L1267	√	×	√				
T1398	√	√	√				
I1399	√	√	√				
D1411	×	×	×				
K1412	√	×	×				
K1415	√	×	×				
S1419	√	×	×				
V1428	√	√	√				

Table S10: Presence/Absence of the **NEUTRAL** mutations on the Ramachandran plot of the WT structures of the three considered templates. Only high quality parts of the models have been considered for the RAMPAGE analysis. Legend: √= residue present; ×=residue not present and ×◦= residue not present, but its first neighbor yes.

5 Graph kernels and Dominant Set further results

In this section we complete the presentation of the kernels results by showing the similarity matrices and the dominant set results not included in the main paper. We recall that each similarity matrix has rows and columns numbered in the range 0-84, which are the mutations ids already shown in the main paper: according to the ids list, note that ids 0–29 are relative to pathogenic mutations while ids 30–84 identify mutations not associated with pain disorders. Each cell (i,j) in a matrix shows the similarity value between the i-th and j-th RINs. The lighter is the cell color the more similar are the two graphs (the main diagonal shows always the lightest color, being the result of the comparison of a graph with itself).

The first analysis examines the role of each interaction separately, with the aim of checking their contribution to the pattern observed in the whole RINs comparison. Figures S4 and S5 show the similarity matrices of the WL kernel for the MOESM3 template and 6A90 template, respectively. Note that interactions H-bond, Van Der Waals and Ionic, taken separately, are in agreement with the pattern observed in the comparison of the whole RINs as shown in the main paper.

Additional insights can be obtained from unsupervised learning techniques applied to the obtained similarity matrices. Figure S6 shows in the (a) and (b) diagrams, the Dominant Set (DS) results for templates MOESM3 and 6J8J, respectively. The diagrams are composed of 85 rows labelled with the ids of considered variants and two columns: one showing the classification resulting from the application of the Dominant Set method and the other one showing the correct classification, which is known, and distinguish between pain related (**PAT**) mutations (dark color) and neutral (**NEUTRAL**) variants (yellow). The DS classification of the two templates are in line with their WL kernel results. In particular, template MOESM3 obtains a good classification: among the pathogenic mutations only two are misclassified. Concerning template 6J8J, since the kernel is not able to discriminate the two clusters, it is not a surprise that also DS does not show a good result.

6 Considering other human NaV1.7 templates

We considered three further templates that stem from the paper by Xu et al (Cell 2019) and refer to the following structures that model only the voltage-sensor domain II (VSD2) of Nav1.7. The PDB entry 6N4Q represents the protein in the activated state and is in complex with a spider toxin; the PDB entry 6N4R represents NaV1.7 in deactivated state, again in complex with a spider toxin. Neither of these two cases refer to the closed state and hence cannot be compared with those of the present study. Instead, the PDB entry 6N4I is is a chimeric structure that model the NaV1.7 protein in closed state. As we further elaborated below, however, the quality of this template is significantly lower

compared to the other human template 6J8J and it cannot be used efficiently in our computational pipeline. We start by superimposing the two structures in Figure S7 with 6J8J in cyan and 6N4I in gold.

The 6N4I template is obtained via X-ray crystallographic diffraction with 3.54 Å resolution and is formed by only 245 amino acids that belong to the second domain. Such structure has been then replicated by symmetry in order to model the other three domains and complete the structure of the sodium channel. Instead, the 6J8J template represents 1193 amino acids out of the nearly 2000 of the whole protein sequence. We compared the two templates 6N4I and 6J8J and calculated their Root Mean Squared Deviation (RMSD): while the RMSD between 128 selected and pruned amino acids is 1.25 A, the total RMSD is larger that 35 A.

This notwithstanding, we have used Swiss-model to perform homology modelling using the WT sequence and the 6N4I template, in a way akin to that we did for the 6J8J template in the original manuscript. The resulting structure is depicted in Figure S8.

As noticeable, the geometry of the sodium channel is quite different with respect to the original template at variance with what was happening when using the 6J8J template. We ascribed this result both to the significantly lower quality of the original crystal of the 6N4I deposited structure and to the much lower number of represented amino acids. Hence, the structure of 6N4I is clearly insufficient to be used for homology modeling.

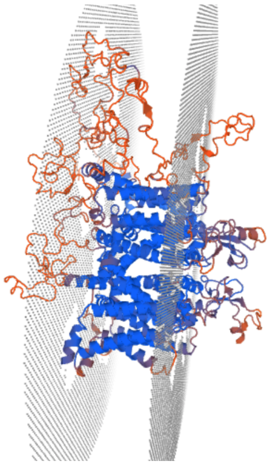

(a) Structure

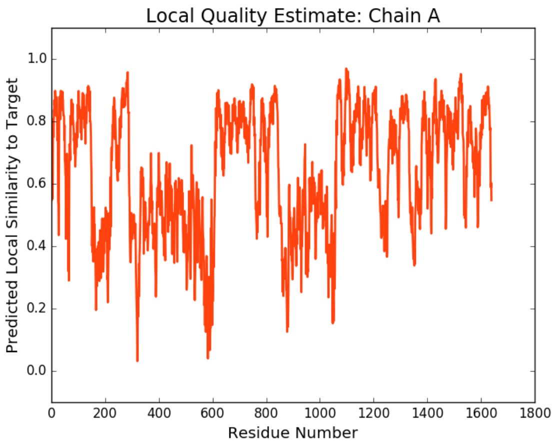

(b) Quality values along the peptide sequence

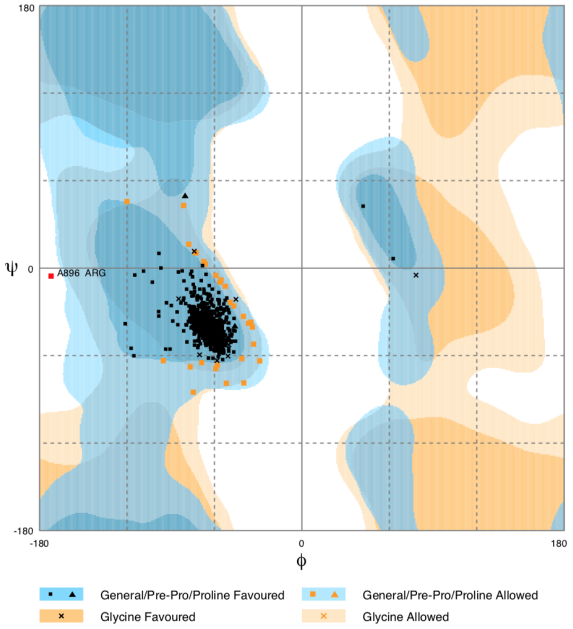

Number of residues in favoured region (~98.0% expected) : 667 (95.8%)
Number of residues in allowed region (~2.0% expected) : 28 (4.0%)
Number of residues in outlier region : 1 (0.1%)

(c) Ramachandran plot analysis

Figure S1: Quality results for MOESM3 Wild Type.

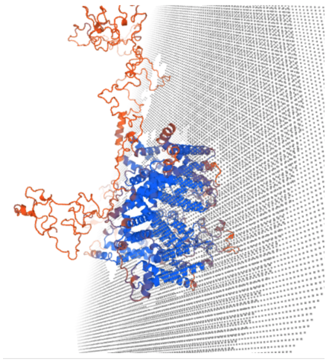

(a) Structure

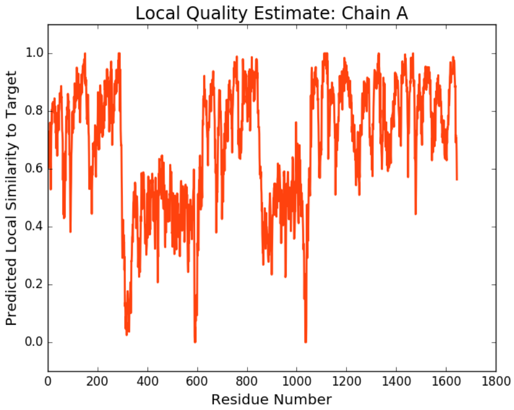

(b) Quality values along the peptide sequence

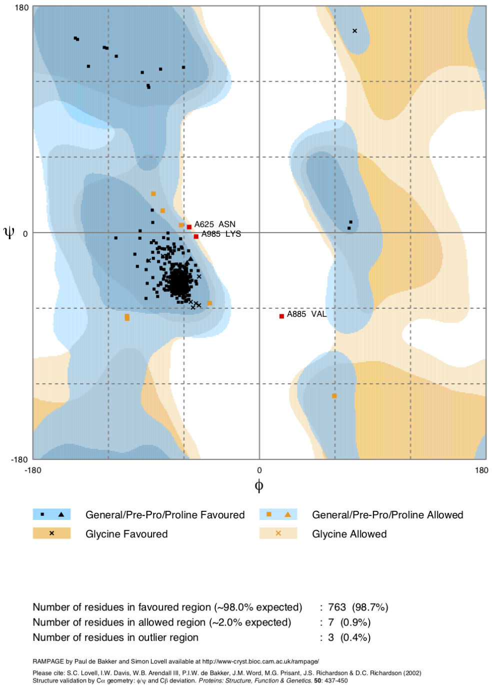

(c) Ramachandran plot analysis

Figure S2: Quality results for 6J8J Wild Type.

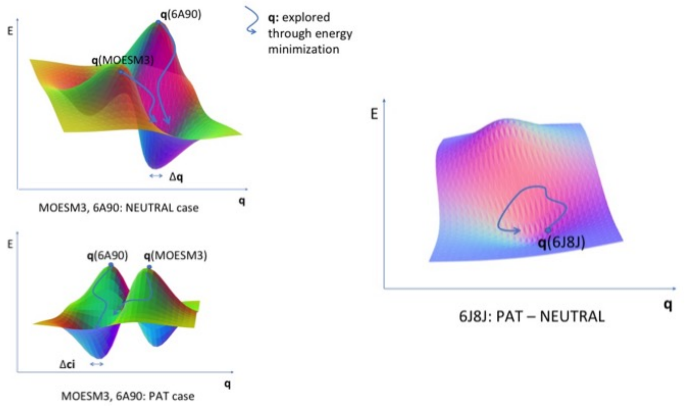

Figure S3: Hypothetical energy landscape explored in the energy minimization step for each template.

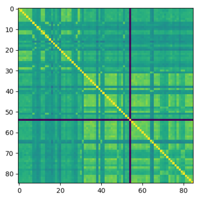

(a) H-bonds

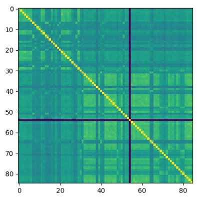

(b) Van der Walls

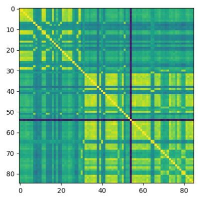

(c) Ionic

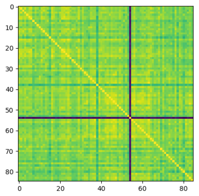

(d) $\pi - \pi$ stack

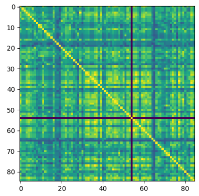

(e) π-cations

Figure S4: Similarity matrices of the Weisfeiler-Lehman (5 iterations) kernels applied to RINs resulting from MOESM3 template with separated interactions

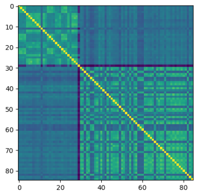

(a) H-bonds

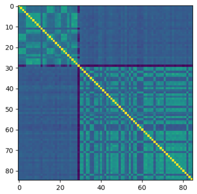

(b) Van der Walls

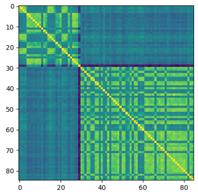

(c) Ionic

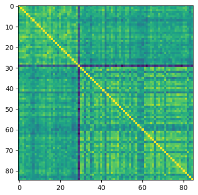

(d) $\pi - \pi$ stack

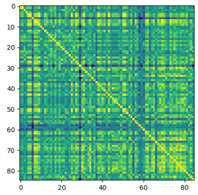

(e) π cations

Figure S5: Similarity matrices of the Weisfeiler-Lehman (5 iterations) kernels applied to RINs resulting from 6A90 template with separated interactions.

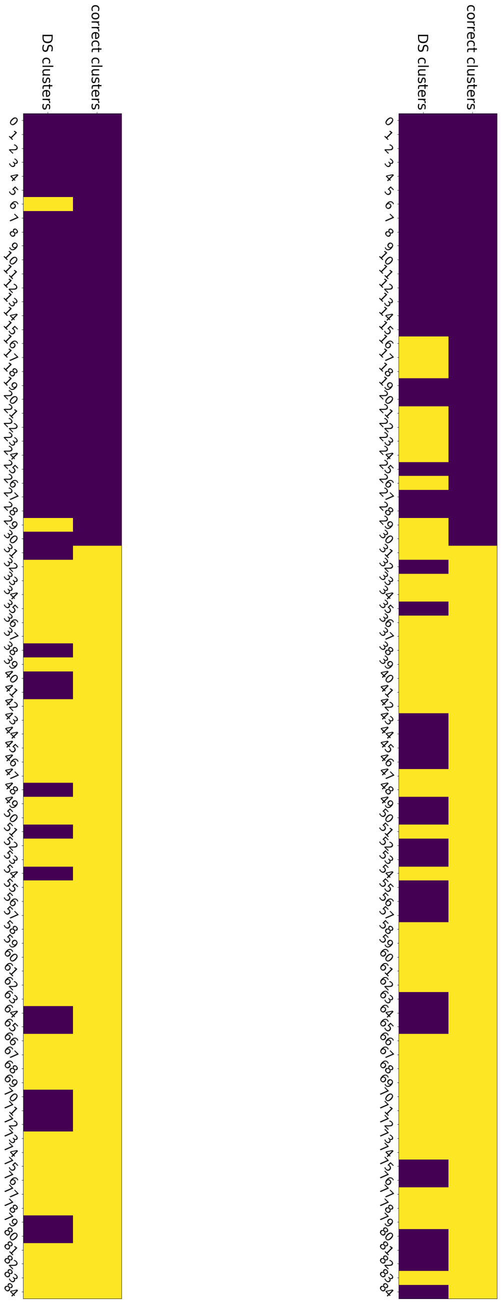

(a) MOESM3 (b) 6J8J

Figure S6: DS classification (one iteration) for templates MOESM3 (a) and 6J8J (b) templates with WL kernel.

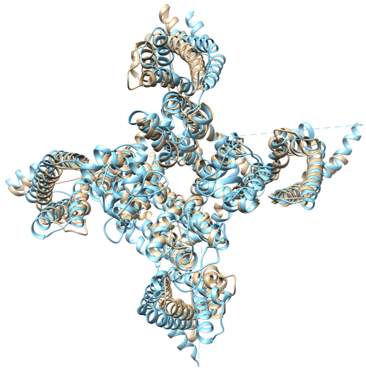

Figure S7: 6J8J (in cyan) and 6N4I (in gold)

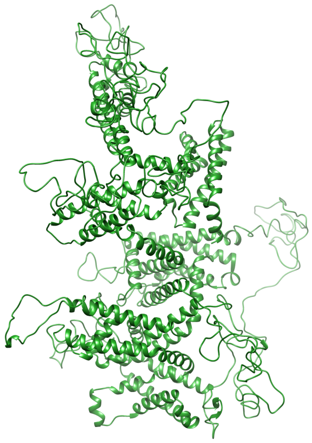

Figure S8: 6N4I: homology modeling resulting structure for the WT sequence
